# Nursing students’ satisfaction of the clinical learning environment: a research study

**DOI:** 10.1186/s12912-016-0164-4

**Published:** 2016-07-19

**Authors:** Evridiki Papastavrou, Maria Dimitriadou, Haritini Tsangari, Christos Andreou

**Affiliations:** Department of Nursing, School of Health Sciences, Cyprus University of Technology, Limassol, Cyprus; Statistician, Department of Economics and Finance, School of Business, University of Nicosia, Nicosia, Cyprus

**Keywords:** Nursing education, Mentorship, Clinical environment, Nurse Teacher, Satisfaction

## Abstract

**Background:**

The acquisition of quality clinical experience within a supportive and pedagogically adjusted clinical learning environment is a significant concern for educational institutions. The quality of clinical learning usually reflects the quality of the curriculum structure. The assessment of the clinical settings as learning environment is a significant concern within the contemporary nursing education. The nursing students’ satisfaction is considered as an important factor of such assessment, contributing to any potential reforms in order to optimize the learning activities and achievements within clinical settings.

The aim of the study was to investigate nursing students’ satisfaction of the clinical settings as learning environments.

**Method:**

A quantitative descriptive, correlational design was used. A sample of 463 undergraduate nursing students from the three universities in Cyprus were participated. Data were collected using the Clinical Learning Environment, Supervision and Nurse Teacher (CLES + T).

**Results:**

Nursing students were highly satisfied with the clinical learning environment and their satisfaction has been positively related to all clinical learning environment constructs namely the pedagogical atmosphere, the Ward Manager’s leadership style, the premises of Nursing in the ward, the supervisory relationship (mentor) and the role of the Nurse Teacher (*p* < 0.001). Students who had a named mentor reported more satisfied with the supervisory relationship. The frequency of meetings among the students and the mentors increased the students’ satisfaction with the clinical learning environment. It was also revealed that 1st year students were found to be more satisfied than the students in other years.

**Conclusion:**

The supervisory relationship was evaluated by the students as the most influential factor in their satisfaction with the clinical learning environment. Student’s acceptance within the nursing team and a well-documented individual nursing care is also related with students’ satisfaction. The pedagogical atmosphere is considered pivotal, with reference to students’ learning activities and competent development within the clinical setting. Therefore, satisfaction could be used as an important contributing factor towards the development of clinical learning environments in order to satisfy the needs and expectations of students. The value of the development of an organized mentorship system is illustrated in the study.

## Background

Mobility of health care professionals is a growing phenomenon worldwide and in the case of Europe several policies have been developed in order to harmonize nursing education in the European countries. Thus, the duration and the content of both theoretical teaching and practice in the clinical areas is explicitly regulated by the European directive2013/55/EU [1] that recommends that 50 % of the total duration of the undergraduate nursing education need to include clinical practice in order to get a registration as a nurse. All the educational and learning activities during the clinical placements of nursing students could be compound into a broader concept, the Clinical Learning Environment (CLE).

The CLE is an interactive network of forces within the clinical setting that influences learning outcomes [2]. It includes everything that surrounds students and affects their professional development in the clinical setting. There is considerable evidence supporting the CLE as extremely beneficial in familiarizing students with clinical judgment and decision–making [3], in stimulating their critical thinking [4], in challenging students to recognize the consequences of their mistakes [5], and in exposing them to various socio-cultural, biological, psychological and mental aspects of patients’ care [3]. The CLE is the place where the theoretical components of the curriculum can be integrated with the practical and transformed into professional skills and attitudes within an emotionally safe environment [6]. However, from the nursing students’ point of view, CLE is “the most anxiety-provoking component of nursing education” [7] as they have to satisfy a dual role, that of the learner and that of the worker. The ongoing changes in health care needs together with the shift in nursing education to academic levels, have transformed students’ clinical experiences from “learning by doing” to evidential oriented learning. However, not all the clinical settings are conducive to students’ learning outcomes or contributing to their competencies’ development [8].

Within this context, it is not surprising that the quality of clinical preparation of students has been systematically debated since 1980, in order to reach an optimal level of clinical learning achievements [9]. In many recent studies, students’ satisfaction has been consistently identified as an important factor of a “good” clinical learning environment [10].

Although the CLE has been investigated in various educational respects, there is a scarcity of studies exploring the nursing students’ point of view from the standpoint of their satisfaction with the CLE on a worldwide basis. This current study aims to explore Greek Cypriot undergraduate nursing students’ level of satisfaction with the CLE in hospital settings. Students view hospital practice areas as more meaningful and educative because they provide them with opportunities of clinical practice and linking the theoretical aspect of their studies [11]. This will provide important feedback for clinical education and potential curriculum revisions [11–13]. To this end, the current study is intended to contribute empirical evidence as to the existing evidence within the relevant, international literature thus allowing potential comparisons of the nursing students’ satisfaction nationally and across countries.

“The attainment of certain outcomes of a clinical placement may be enhanced by modifying the CLE in ways that make it more congruent with the environment preferred by students” [14]. For the present, there is a lack of a clear and commonly accepted definition of what contributes to nursing students’ satisfaction with the CLE. This may be due to different conceptual approaches that occurred across the relevant studies and due to the fact that the students’ satisfaction seems to depend on various dimensions of teaching and learning in clinical settings [15].

Students’ satisfaction is a complex and multifactorial issue [16]. Relevant studies revealed positive links between students’ satisfaction and the quality of nursing care [10, 17], the ward’s pedagogical atmosphere and leadership style [3, 12, 13] the sense of belonging [18], the peer support [19] and the motivation level [20]. On the other hand, students’ supervision and the relationship among the nursing students and mentor [8, 20] or nurse teacher (NT) [3, 21] have been considered as the most noteworthy elements for the effectiveness of the CLE with reference to nursing students’ learning and professional development.

Previous studies [3, 22] highlighted the importance of the interpersonal relationships on the effectiveness of the clinical experience and student satisfaction. Patients [23] peer [19] ward staff [22] mentor and clinical teacher [21] are the major stakeholders involved in that experience-rich, supportive relationship. Students’ positive clinical experiences are more likely to be related to how valued and supported students feel than the physical aspects of a placement [24]. High levels of satisfaction have been reported when students had someone to ensure that their learning needs were addressed, when the clinical staff were well briefed [11] when the students were treated with respect and appreciation [25] as well as being included as part of the health care team [26]. Other issues on which students expressed satisfaction concerned effective levels of mentor expertise and guidance [8, 25, 27], continuous feedback on their professional performance [26], frequent clinical conferences with their mentor and NT [20, 22], and the concurrence of clinical practice with theory [10]. However, the degree of satisfaction appeared to be influenced by the unique organizational atmosphere of each nursing ward [28], the duration of clinical placement [13], the years of study [17] and educational supervision [22].

Efforts at producing a high quality of CLE have recently been focused on creating a pedagogical atmosphere, and strengthening the connection between university classwork and placement experience by means of adoptive supervision models [21, 22]. The two models primarily used in European countries involve: a) the mentor or preceptor (these terms are used interchangeably) and represent an experienced clinical nurse affiliated in the university, focused on translating knowledge in skillfulness and b) the NT who is employed by the educational institution acting as a liaison, confirming theory –practice continuum.

These clinical learning models were also adopted by the nursing programs of all the Universities in the Republic of Cyprus. Usually they include clinical skills of about 90 ECTS (European Credit Transfer System) according to the European and National standards [29] and the duration of clinical practice increases according to the year of study and the different learning objectives that have to be achieved. The supervisory role in the clinical settings is undertaken mainly by named mentors who are supported by members of the academic staff of each University. Mentors’ work is considered very important in supporting the professional development of the nurse students and the assessment of their “competencies” [30]. For this reason, the chosen mentors attend a mandatory two days seminar in order to understand nursing curriculum and help students to get the best from practice placement. The mentor\student ratio is 1:5, both are supernumerary and this gives the mentor the opportunity to be more student-oriented and devote his\her time exclusively to the needs of the students.

Although there is a lack of a conceptual and theoretical background describing the nursing students’ satisfaction, there is evidence that it could be conceptually clarified and measured within the context of an internationally accepted tool, specifically the Clinical Learning Environment, Supervision and Nurse Teacher (CLES + T) [31]. In this context, the nursing students’ satisfaction was described within five distinctive constructs. The *ward’s pedagogical atmosphere* includes the teamwork and the personnel’s interest in students’ learning needs. The *supervisory relationship* constructs that stands for the sense of trust, student/mentor equality and continual feedback. The third construct reflects the *ward’s leadership style* representing the relationships between the ward managers, the staff and the students. The *premises of nursing on the ward* refer to the organization of the nursing care and the *NT’s role in clinical practice* is defined as the nurse teacher’s ability to minimize the theory-practice gap.

## Methods

### Aim and research questions

The study aimed to investigate nursing students’ satisfaction with the clinical learning environment. With this in mind, the following research questions were posed:

1. What is the level of Cypriot nursing students’ satisfaction with the CLE?
2. If any, what is the relationship between the students’ satisfaction and some of their personal data (e.g., year of study, type and frequency of supervision)?
3. If any, what is the relationship between the students’ satisfaction and the five dimensions of the clinical learning environment, as defined within the CLES + T?

### Research instrument

The CLES + T scale was developed and validated by Saarikoski et al. [31] and adapted in many languages as a self-report questionnaire designed to measure the nursing students’ perceptions of their satisfaction of the CLE. The questionnaire consists of 34 items classified into 5 dimensions: pedagogical atmosphere on the ward; supervisory relationship; leadership style of the ward manager; premises of nursing on the ward; role of the NT in clinical practice [31]. Respondents are asked to score their perception of each item on a 5-point Likert-type scale ranging from “very dissatisfied” to “very satisfied”. The instrument has been adapted to the Greek language [13, 17] reporting reliable and valid measures with scales’ Cronbach’s alpha values ranging 0,82–0.96 [20].

For the purpose of the current study, the CLES + T Greek version was used alongside a questionnaire on demographic data referring to the university, the gender, the age and the participants’ educational level. Demographics also included learning-teaching characteristics such as the hospital and the ward type, the clinical placement length, the frequency of the weekly meetings with the NT, the use of e-contact with the NT during clinical placement and the motivational level of the clinical setting. The satisfaction with the CLE was examined by one general question in a 5-point Likert-scale.

### Sampling and data collection

In the Cyprus Republic there are four universities leading to a bachelor’s degree in nursing, one state and three private institutions. One private university refused to participate in the study, so only three universities were included. The total nursing student population of the 3 universities were 664 individuals. The inclusion criteria for students’ participation were: (1) the students’ informed consent and (2) practicing in hospitals and not in community settings. The questionnaires were personally administered to the students during the last nursing laboratory lesson towards the end of the academic year 2012–13. Nursing students were provided with information with regard to the purpose of the study, the anonymity of the collected data and the voluntary nature of their participation. From the total population, 463 questionnaires were returned, giving a response rate of 70.3 %. Four of the questionnaires were removed from the data analysis as they were not properly completed and so the final sample consisted of 463 nursing students.

The research proposal was approved by the National Bio-Ethics Committee and permissions to access participants granted from the universities’ authorities. Permission to use the CLES + T was obtained by the authors.

### Data analysis

Descriptive statistics were used to calculate frequencies, means and standard deviations from the demographic data. The reliability of the data was estimated with Cronbach’s alpha. Taking into account the data deviation from normality, non-parametric inferential statistics were selected. The correlation analyzes between the students’ satisfaction and the five constructs of the CLE were performed with Spearman’s rho correlation coefficient. Chi-square tests were also used to examine the relationship between the nominal scales of students’ satisfaction and their demographic data as well as regarding students’ relationship with their mentors. In order to examine the relationship between the ordinal scale of students’ satisfaction with students’ demographic data and relationship with their mentors, multinomial logistic regression was used. The simultaneous entering of all the independent variables in the model has the advantage of reducing Type I errors. However, individual Chi-square tests were also performed for additional insight into the relationships.

## Results

The results showed high internal consistency for the total CLES + T (Cronbach’s alpha = 0.95) and for each of the five dimensions, ranging from 0.81 (“premises of nursing care in the ward”) to 0.97 (“supervisory relationship”). Regarding the sample’s demographics, 38.7 % were males and 61.3 % females, with age ranging from 18 to 34 years, with a mean of 21.08 years and standard deviation 2.23 years. 149 participants studied in private universities and 318 at the public university (Table 1).

**Table 1:** Demographic data (*n* = 463)

Variable	f	f/n (%)
Type of University		
Private university	149	32.2
Government university	314	67.8
Gender		
Male	179	38.7
Female	284	61.3
Year of study		
1st year	111	24.0
2nd year	110	23.8
3rd year	121	26.1
4th year	121	26.1
Type of nursing ward of last clinical placement		
Geriatrics	2	0.4
Surgical	81	17.5
Gynecology	6	1.3
medical	88	19.0
Pediatrics	65	14.1
Psychiatric	40	8.7
Other	180	39.0
Did any changes take place during your placement		
No	266	58.3
Yes	79	17.3
I can’t evaluate	111	24.3
Type of the hospital in which clinical placement was held		
General Hospital	427	92.2
Specialized care center	7	1.5
Outpatient department	13	2.8
Other	16	3.5
How many times did you meet NT during the latest clinical placement		
Never	32	6.9
1–2 times	82	17.7
3 times	32	6.9
Often	316	68.4
Did you use e- communication tools with your NT during placement		
I have never used	274	59.4
1–3 times	114	24.7
4–6 times	30	6.5
Moreoften	43	9.3

The mean score for the total sample of nursing students’ satisfaction was estimated at 4.1, supporting the contention that students perceived the CLE as “very good”. The relationship between the five CLES + T dimensions and the question that measured the general satisfaction of student was examined. Spearman’s rho correlation coefficient was significant between the overall satisfaction and all of the five dimensions (*p* < 0.001) *(*Table 2). Similarly, the overall students’ satisfaction was positively correlated with all the items (*p* < 0.001) (Table 3)

**Table 2:** Bivariate Correlations between the “total satisfaction” items with the CLES + T dimensions(*n* = 463)

Totalsatisfaction	Scale	Pedagogical Atmosphere	Leadership style of Ward Manager	Premises of Nursing in ward	Supervisory Relationship	Nurse Teacher role
Spearman’s Corr.coefficient	0.610^**^	0.521^**^	0.388^**^	0.385^**^	0.550^**^	0.432^**^
*p*-value	< 0.001	< 0.001	< 0.001	< 0.001	< 0.001	< 0.001

**Correlation is significant at α = 0.01

**Table 3:** Correlations between all the CLES + T items and nursing students’ satisfaction (*n* = 463)

CLES + T items	Corr. Coefficient	*p*-value
Pedagogical atmosphere (PA)		
PA1 The staff was easy to approach	.429	< 0.001
PA2 I felt comfortable going to the ward at the start of my shift	.265	< 0.001
PA3 During staff meetings (e.g., before shifts) I felt comfortable taking part in the discussions	.447	< 0.001
PA4 There was a positive atmosphere on the ward	.471	< 0.001
PA5 The staffs were generally interested in student supervision	.368	< 0.001
PA6 The staff learned to know the students by their personal names	.194	< 0.001
PA7 There were sufficient meaningful learning situations on the ward	.372	< 0.001
PA8 The learning situations were multi-dimensional in terms of content	.371	< 0.001
PA9 The ward can be regarded as a good learning environment	.476	< 0.001
Leadership style of the ward manager (WM), Premises of Nursing on the ward (NC)		
WM10 The WM regarded the staff on his/her ward as a key resource person*	.344	< 0.001
WM11 The WM was a team member*	.279	< 0.001
WM12 Feedback from the WM could easy be consider a learning situation*	.352	< 0.001
WM13 The effort on individual employee was appreciated*	.302	< 0.001
NC14 The ward nursing philosophy was clearly defined*	.316	< 0.001
NC15 Patients received individual nursing care*	.317	< 0.001
NC16 There were no problem in the information flow related to patients’ care*	.244	< 0.001
NC17 Nursing Documentation (e.g., nursing plans, daily procedures etc.) was clear*	.363	< 0.001
Supervisory relationship (SR)		
SR18 My supervisor showed a positive attitude towards supervision*	.440	< 0.001
SR19 I felt that I received individual supervision *	.469	< 0.001
SR20 I continuously received feedback from supervisor*	.500	< 0.001
SR21 Overall I am satisfied with the supervision I received*	.568	< 0.001
SR22 The supervision was based on a relationship of equality and promoted my learning*	.505	< 0.001
SR23 There was a mutual interaction in the supervisory relationship*	.495	< 0.001
SR24 Mutual respect and approval prevailed in the supervisory relationship*	.508	< 0.001
SR25 The supervisory relationship was characterized by a sense of trust*	.509	< 0.001
Role of the nurse teacher (NT)		
NT26 The NT was capable of integrating theoretical knowledge and everyday practice*	.333	< 0.001
NT27 The NT was capable of operational sing the learning goals of this placement*	.323	< 0.001
NT28 The NT helped me to reduce the theory-practice cap*	.317	< 0.001
NT29 The NT was like a member of the nursing team*	.309	< 0.001
NT30 The NT was able to give his or her expertise to the clinical team*	.352	< 0.001
NT31 The NT and the clinical team worked in supporting my learning*	.362	< 0.001
NT32 The meetings between myself mentor and NT were comfortable experience*	.418	< 0.001
NT33 In our common meetings I felt that we are colleagues*	.416	< 0.001
NT34 Focus on meetings was in my learning needs*	.349	< 0.001

*Correlation is significant at *p* = 0.01

.

Multinomial logistic regression showed significant relations between students’ satisfaction and demographics, as well as with the student’s relationships with their mentors (Table 4). The results showed that significant differences existed in terms of year of study (*p* = 0.01), the frequency of meetings with NT (*p* = 0.02), the frequency of supervision with mentor (*p* = 0.05), the method of supervision (*p* = 0.01), mentor or NT (*p* < 0.001) and the motivation (*p* < 0.001). Specifically, the first year students had the highest satisfaction level compared to the other years (2nd to 4th). Those students who reported that they met with their NT at least three times per week had a higher level of satisfaction in comparison to those that met only once or twice a week. The highest satisfaction was found among those students who had a named mentor with whom their relationship was effective, while the lowest was reported by those who did not have a named supervisor. Students who had frequent meetings with their mentor had a higher level of satisfaction compared to those who did not have any meetings at all, or had very rare meetings (e.g., once or twice in total per week). Those who answered that the most important person was the NT had the lowest satisfaction, compared to those who placed emphasis for their supervision on “mentors” or “both” the NT and a mentor. Students who had no motivation had the lowest satisfaction, followed by those who had some motivation. The most satisfied nursing students were those who had high levels of perceived motivation by the CLE. It should be noted that individual Chi-square tests showed the same significant relations, (notice that the relation with frequency of supervision with mentor was highly significant, with *p*-value = 0.01), while two additional variables were found to be significant, namely University (*p* = 0.04) and type of nursing ward (*p* = 0.02), where the results revealed that nursing students from the private universities were found to be less satisfied than those from the state university, and students practicing in paediatrics units had the lowest satisfaction compared to other nursing wards.

**Table 4:** Multinomial Logistic regression for the examination of the relation between nursing students’ satisfaction and personal data (*n* = 463)

Item	Chi Square	df	*p*-value
Q1: University	1.315	4	0.85
Q2: Age	44.751	44	0.44
Q3:Sex	1.340	4	0.85
Q4: Year of study*	27.917	12	0.01
Q5: Type of nursing ward of the clinical placement	3.854	16	0.99
Q6: Changes in the ward during the clinical placement	5.201	8	0.73
Q7: Hospital type	14.369	12	0.27
Q10: How many times did you meet the NT during the last clinical placement*	25.011	12	0.02
Q11: Use of e-communication tools with the NT during placement	11.488	12	0.48
Q12: Occupational title of the mentor	8.552	12	0.74
Q13: Methodof supervision (with mentor)*	21.493	8	0.01
Q14: Frequency of separate private supervision with mentor *	25.764	16	0.05
Q15: Mentor orNT *	28.277	8	< 0.001
Q16: Motivation *	71.561	8	< 0.001

*Relation is significant at α = 5 %

In the examination of the relationship between the method of supervision and the satisfaction, the six method levels were grouped into three subcategories to facilitate presentation and interpretation. The first subcategory (so called unsuccessful supervision) included the following characteristics: the student did not have a named supervisor, a personal supervisor was named, but the relationship did not work or the named supervisor changed during the course of training. The second subcategory (so called group supervision) referred to cases where the supervisor had several students and satisfaction varied according to shift or location. The third subcategory (so called successful supervision) included students who had a named mentor and their relationship was effective. Taking eight degrees of freedom and *p*-value smaller than 0.001, significant differences were revealed across the satisfaction level and the supervision method. Further examination of data showed that the highest satisfaction level was found for those with successful supervision (mean satisfaction = 4.11), followed by those with group supervision (mean satisfaction = 3.86), and the least satisfied were students with unsuccessful supervision (mean satisfaction = 2.61).

## Discussion

The positive learning experiences revealed in the current study correspond to the supervisory relationship in combination with the frequency of individual meetings, the presence and support of the NT and the sense of “team spirit” in a well-organized nursing care environment. These findings are in agreement with previous findings of the Cypriot studies [13, 17, 20] as well as other relevant studies [11, 12, 22]. The current results generally showed that nursing students’ satisfaction with their practice environment was significantly related to all of the five CLES + T dimensions: pedagogical atmosphere, ward manager leadership style, premises of nursing on the ward, supervisory relationship and the role of the nurse teacher (*p* < 0.001). The students’ satisfaction was also found to be positively related to all the individual items of the factors comprising the learning environment in clinical settings. Although the evidence on student satisfaction from learning in the practice environment is limited, efforts have been made to improve the quality of clinical placements in Cyprus and elsewhere [19]. Therefore our results can be partly explained by the NT involvement through regular visits and collaboration with the mentors. The NT also acts as a supporter to the mentor and together they organize students’ clinical learning, so that each has the opportunity to participate in the learning situation.

The mentorship relationship was evaluated by the students as the most influential factor for their satisfaction, a similarity that was echoed across earlier studies using the CLES + T [12, 13, 20, 22]. Students with a named mentor (successful supervision) reported themselves as more satisfied with the dimensions concerning the mentorship relationship, suggesting the idea that students’ experiences of their relationships and of being treated as unique individuals are supporting agents for their learning and increase their sensitivity to the patients’ needs [32]. This finding supports what was reported by one European study [32] and confirms the nursing students’ preference towards one-to-one supervision. This could also be considered a further argument supporting the individual learning approach of the nursing students, a long accepted axiom of adult learning. In accordance with the study of Dobrowolska et al. [33] in comparing the mentor’s role in 11 EU and non- EU countries, the type of mentor who are staff nurse working in the clinical area focus mainly on developing clinical competences in the students, although is accused of lacking teaching experience and pedagogical education. However, having a named mentor during the clinical placement might be disadvantageous if the mentorship relationship “doesn’t work”, as student would then have to deal with issues of negativity and prejudice [34].

In addition to this concern, it was also argued that a successful mentorship might be depended on student preparedness, readiness and willingness to learn [35]. Finally, in line with other relevant studies, satisfaction from mentorship supervision was found to be differentiated according to the clinical settings [12], the method of supervision and the frequency of meetings [13, 20].

Regarding the academic year, the first year students reported the highest satisfaction compared to other, later years. This was reported likewise within other relevant studies [10], which showed satisfaction to decline as students progressed through the program. A probable explanation for that finding might be the fact that the learning objectives and activities differed in the academic progress [19]. The first year students felt high levels of physical and mental stress due to their limited capacities in terms of fundamental clinical skills [19]. Therefore, mentoring was recommended as a teaching strategy to minimize anxiety and to help novice students to deal with the feelings of unpreparedness [7]. On the other hand, there is evidence that the third year students expressed higher confidence in clinical knowledge and skills, and tend to focus on leadership and guidance [19]. Henderson et al. [36], have explicated that third year students’ satisfaction is associated with involvement, personalization, greater motivation and commitment towards their learning needs and the patient care.

Also, the frequency of meetings with the NT and mentor in relation to the satisfaction level was demonstrated. Even though the two models of supervision had different perspectives, the nursing students expressed their satisfaction from the meetings with both the NT and the mentor. Students’ satisfaction is also shown in the statement declaring that “*The common meetings between myself mentor and NT were a comfortable experience”*, suggesting that NT presence in clinical area, as a member of academic staff, is useful in order to monitor and guide through the medium of the mentor–student relationship and to act as a learning advocator [37]. The NT is considered to be the person who is responsible for the careful planning of the clinical placement, and therefore regular visits enhanced students’ clinical experience because those visits ensured that student educational goals were successfully achieved in a timely fashion [25, 37]. In contrast, students may feel abandoned when they have no or few visits, especially when they were placed in new environments [37]. In terms of unfamiliar nursing team and organizational philosophies, they appreciated the presence of the NT in order to give ongoing guidance to the ward staff with regard to the anticipated performance level at the student’s particular stage of learning [38]. Besides, the NT’s role as educator with a clinical background and working as a liaison between the university and clinical settings has been documented by several relevant studies as effective, especially in stressful situations [22]. However, the weak correlation (*r* = 0.381 *p* < 0.001) between students’ satisfaction and both of the CLE dimensions *Role of the NT* and *leadership style of the ward manager,* showed that the supervision models of ward placements may have led ward managers not to place students’ education within the priorities of the ward [35]. This may indicate a conflict of tasks and priorities between the needs of healthcare and the goals of the universities’ personnel. However, the fact remains that the NT’s presence is influential on the nursing staff to involve themselves in the students’ learning process [37].

Another finding that is congruent with previous studies [20] is the lower satisfaction reported by those participants who answered that the most important person to understand nursing practice was the NT compared to those who said “mentor” or “both”; suggesting that students evaluate both roles positively. However, the mentor’s role seemed to prevail in the fulfillment of prerequisite clinical competencies and advance the socialization process in clinical settings [12, 24]. The mentor was perceived to be essential for ensuring clinical safety while students developed their scope of practice [39]. However, the mentor has the dual role of an educational supervisor and an assessor. He was therefore perceived to violate his educational expectations as well as those of the students. As noted in Price et al. [37] study, NT’s clinical visits have to be encouraged so as to develop well-informed and confident mentors, ensuring continuity between the idealized class world and the practice reality.

Finally, the strong and significant correlation found between the *pedagogical atmosphere* and *premises of nursing care* indicated that students’ satisfaction is higher when they are actively involved in individual patient care with clear information flow and clear documentation of nursing care within a welcoming and educationally structured environment. This is in line with the findings of previous studies [20, 34], confirming that the CLE is related to the quality of nursing care and patient contact [17]. It is accepted, though, and confirmed by other European studies that a task-oriented approach to nursing care is considered a barrier to students’ learning [35]. In the Papp et al. [25] study, students associated the quality of clinical practice with the quality of mentorship and the quality of patient care. The feeling of being welcome as students was manifested by the way the nursing staff approached them. Specifically, a notable observation of the current study, which concurs with a previous Cypriot study [20] is that the significantly lower satisfaction with item six “*The staff learned to know the students by their personal names”,* which compromised the students dignity as adult learners and implied their status to be “just a pair of hands” [8]. According to the students’ written comments, their dissatisfaction might be attributed to the barriers in accomplishing their learning objectives [11], the constant hurry of staff [26] and the weakness to assert their dual status as supernumerary and as students [35].

Conclusively, the CLES + T scale could be considered useful in exploring nursing students’ satisfaction with their clinical experiences and the supervision with which they are provided. In future studies, satisfaction level could be used as an important contributing factor towards the development and/or reforms of clinical learning environments in order to satisfy the needs and expectations of students.

### Limitations

Some limitations should be noted when drawing firm inferences from the findings of this study due to the relatively short periods of time spent in specific ward environments, specifically from two to three days per week during a period of seven to eight weeks as “short clinical rotations”. These may not provide sufficient time to build mutual understanding and familiarity within the specific CLE. A longitudinal approach will be very helpful in assessing students’ satisfaction with the CLE from the standpoint of the novice as younger students to experience as older students. Of course a mix methodology similar to other relevant studies [11, 22] could provide further insights of the students’ qualitative responses as regard their views and/or perceptions of the CLE. However students’ satisfaction could not be considered as the only measurement to assess the CLE’s impact on students’ learning and development. Within such mix methodology there might emerge other interesting and unknown variables. Yet despite the limitations of the current study, our results are much in accordance with recent relevant studies conducted across EU countries. Nevertheless, addressing the lack of a relevant grounded theoretical or conceptual model for this field would be of value. Nursing students represent the future nursing workforce, thus nursing education is an important investment for the quality of the provided clinical nursing care. During a pre-registration program students are systematically prepared to reach the minimum standards of ‘competencies’ on knowledge, skills and attitudes, during their clinical learning in order to be certified for their professional capability.

## Conclusions

The need for evaluating students’ satisfaction with their practice environment is associated with two important issues of nursing workforce: the competency of graduates and students’ retention. In the context of the current study, Cypriot nursing students were found to be highly satisfied with the CLE and this was related to the level of motivation and the nursing care delivery, the supervisory relationship with the mentor and NT’s role in clinical practice. However, students’ satisfaction was found to change according to the level of study, indicating that learning needs and expectations differentiate as student coming up the“ladder of competence” indicating that clinical supervision and support need to be tailored according to the their individual needs.

The current study illustrated the value of the development of an organized mentorship system. This was viewed by the participant nursing students as one of the most important variables in their clinical learning and their satisfaction with the CLE. Student’s acceptance within the nursing team and the organization of nursing care impact students’ satisfaction.

Under current economic distress, there is a need to re-clarify the potential roles of all parties involved in students’ clinical learning so that adequate preparation will be made to meet educational objectives. Also, findings support the need to encourage both the nursing staff and the mentors in order for theoretical knowledge to be effectively transferred into clinical practice, thus minimizing the gap between the theoretical ideal and the reality of the clinical world. However, the findings across relevant studies presented an ensemble of factors that impact nursing students’ satisfaction of the CLE. Further investigation of the field is therefore recommended.

## Abbreviations

CLE, Clinical Learning Environment; CLES + T, Clinical Learning Environment, Supervision and Nurse Teacher; *NT, Nurse Teacher*
